# Impact of *PTEN* abnormalities on outcome in pediatric patients with T-cell acute lymphoblastic leukemia treated on the MRC UKALL2003 trial

**DOI:** 10.1038/leu.2015.206

**Published:** 2015-08-21

**Authors:** S Jenkinson, A A Kirkwood, N Goulden, A Vora, D C Linch, R E Gale

**Affiliations:** 1 Department of Haematology, UCL Cancer Institute, London, UK; 2 Cancer Research UK & UCL Cancer Trials Centre, London, UK; 3 Department of Haematology, Great Ormond Street Hospital, London, UK; 4 Department of Haematology, Sheffield Children's Hospital, Sheffield, UK

## Abstract

*PTEN* gene inactivation by mutation or deletion is common in pediatric T-cell acute lymphoblastic leukemia (T-ALL), but the impact on outcome is unclear, particularly in patients with *NOTCH1/FBXW7* mutations. We screened samples from 145 patients treated on the MRC UKALL2003 trial for *PTEN* mutations using heteroduplex analysis and gene deletions using single nucleotide polymorphism arrays, and related genotype to response to therapy and long-term outcome. *PTEN* loss-of-function mutations/gene deletions were detected in 22% (*PTEN*^ABN^). Quantification of mutant level indicated that 67% of mutated cases harbored more than one mutant, with up to four mutants detected, consistent with the presence of multiple leukemic sub-clones. Overall, 41% of *PTEN*^ABN^ cases were considered to have biallelic abnormalities (mutation and/or deletion) with complete loss of *PTEN* in a proportion of cells. In addition, 9% of cases had *N-* or *K-RAS* mutations. Neither *PTEN* nor *RAS* genotype significantly impacted on response to therapy or long-term outcome, irrespective of mutant level, and there was no evidence that they changed the highly favorable outcome of patients with double *NOTCH1/FBXW7* mutations. These results indicate that, for pediatric patients treated according to current protocols, routine screening for *PTEN* or *RAS* abnormalities at diagnosis is not warranted to further refine risk stratification.

## Introduction

Approximately 25% of pediatric patients with T-cell acute lymphoblastic leukemia (T-ALL) are likely to relapse with recurrent disease, and this presents a clinical challenge as post-relapse prognosis is poor.^1^ A number of genetic abnormalities, including mutations and copy number changes, have been identified at disease presentation,^2^ and evidence suggests that most leukemic blast cells at relapse are clonally related to those present at diagnosis.^3, 4^ This holds out promise, therefore, that molecular profiling at diagnosis and identification of factors that will predict for relapse could improve outcome by facilitating targeted therapy.^5^ *NOTCH1* and *FBXW7* gene mutations are frequent in both pediatric and adult T-ALL, but although some studies have demonstrated that their presence is associated with a favorable outcome,^6, 7, 8^ others found no significant effect.^9, 10, 11^ However, recent studies have suggested that the impact of *NOTCH1/FBXW7* mutations may be modulated by the presence of coincident abnormalities such as in the phosphatase and tensin homolog (*PTEN*) and *RAS* genes,^12, 13, 14^ and this implies that these genes should routinely be characterized at diagnosis.

PTEN is the main negative regulator of the PI3K/AKT signaling pathway and is implicated in regulating downstream effects of NOTCH1 signaling such as proliferation and survival of T-cell progenitors.^15^ Loss-of-function *PTEN* mutations leading to constitutive activation of AKT were identified in T-ALL cell lines that were resistant to NOTCH1 inhibition with γ-secretase inhibitors,^16^ and a number of studies have now described PTEN loss through mutation and/or genomic deletion in up to 35% of pediatric patients with T-ALL,^12, 13, 14, 17, 18, 19, 20, 21^ although few studies have reported both mutational and copy number status.^12, 14, 18, 21^ The mutations are predominantly clustered in exon 7 and are usually frameshift indels that are predicted to lead to truncated proteins that retain the 5' phosphatase domain but are likely to be unstable.^22^ The genomic deletions generally encompass the whole gene, although microdeletions have recently been reported that result from illegitimate RAG-mediated recombination events.^23^ In addition, the abnormalities can be either monoallelic or biallelic for mutation and/or deletion. RAS is also an important regulator of proliferation through the RAS/RAF/MEK/ERK pathway, and *Kras*-induced mouse models of T-cell leukemia/lymphoma show a strong association with the development of *NOTCH1* mutations.^24, 25^ Activating *N-* and *K-RAS* mutations have been reported in 4–10% T-ALL patients, particularly in early T-cell precursor ALL.^14, 21, 26, 27^

These studies suggest that *PTEN* and *RAS* abnormalities in T-ALL may be suitable candidates as biomarkers and assist in directing targeted therapy, but their prognostic impact in pediatric patients is unclear, particularly in relation to their *NOTCH1/FBXW7* genotype. In general, *PTEN* abnormalities in pediatric patients are associated with non-significantly worse outcome,^12, 13, 23^ although in adult patients, the impact was significantly adverse.^14^ However, whereas one study of pediatric patients found that a trend for adverse impact was not seen in the presence of a *NOTCH1* mutation,^13^ the study of adult patients reported that presence of a *PTEN* or *RAS* abnormality ablated the benefit of a *NOTCH1* mutation.^14^ *RAS* mutations had no impact on clinical outcome in a study of pediatric ALL, but no data were given for the different subgroups.^26^

In our study of 162 patients treated on the Medical Research Council UKALL 2003 trial, we reported that patients with double *NOTCH1* or *NOTCH1* and *FBXW7* mutations (*NOTCH1*±*FBXW7*^Double^) have a very good outcome and should not be considered for more intensive therapy in first remission, even if slow responders or minimal residual disease (MRD) positive after induction therapy.^8^ To determine whether *PTEN* and *RAS* abnormalities impacted on this good outcome, and whether they could refine stratification of cases with single *NOTCH1* mutations (*NOTCH1*^Single^) or wild-type *NOTCH1* (*NOTCH1*^WT^), we investigated *PTEN* and *RAS* genotype in samples from 145 patients treated on this trial and evaluated their impact on outcome in the different subgroups.

## Materials and methods

### Patients and treatment protocol

Diagnostic samples were available from 145 of the 388 (42%) T-ALL patients entered into the UKALL2003 trial, excluding those with bi-phenotypic leukemia or T-cell lymphoma. Ethical approval for the trial was obtained from the Scottish Multi-Centre Research Ethics Committee and informed consent in accordance with the Declaration of Helsinki. The trial opened to patients aged 1–18 years in October 2003. The upper age limit was increased to 20 in September 2006 and 25 in June 2008. Median age of the patients studied was 9 years (range 1–23), 19 were more than 16 years, and only 5 (3%) were more than 18; 111 were male and 34 female. Median follow-up was 6 years 9 months (range, 1–9.8 years).

The trial is registered at http://www.controlled-trials.com under ISRCTN number 07355119. Details of the trial protocol have been published elsewhere,^28^ and an outline is given in Supplementary Figure 1. Patients with National Cancer Institute (NCI) low-risk score at trial entry (age 1–10 years and white blood cell count <50 × 10^9^/l) were allocated to regimen A, those with high-risk score (age >10 years and/or white blood cell count >50 × 10^9^/l) to regimen B. Patients with a slow early response (defined as >25% bone marrow blasts at day 8 or 15 for high- and low-risk patients, respectively) and <16 years of age were assigned to regimen C. At day 29, patients not in morphological remission but with <25% blasts were also reallocated to regimen C; those with resistant disease (>25% blasts) were taken off protocol and those with a rapid early response in morphological remission were assessed for MRD using quantitative polymerase chain reaction (PCR) analysis of leukemia clone-specific rearranged T-cell receptor γ and δ genes.^29^ If MRD of more than 1 leukemic cell in 1000 cells (>10^−3^) was present, patients were classified as MRD-positive and randomized to either remain on their assigned regimen (A or B) or to be reallocated to regimen C. Of the 145 patients included in this study, 13 patients were treated on regimen A, 83 on B and 49 on C.

### Mutation screening and mutant quantification

Full experimental details are given in the Supplementary Data. The entire coding sequence of the *PTEN* gene (exons 1–9) and exons 2 and 3 of the *N-RAS* and *K-RAS* genes were screened by PCR of genomic DNA and heteroduplex analysis on the WAVE DNA Fragment Analysis System (Transgenomic, Glasgow, UK). Samples with abnormal chromatograms were sequenced. Where this showed either multiple or low-level mutations, amplicons were cloned using the TOPO TA cloning kit (Invitrogen, Paisley, UK) and sequenced. Exon 7 mutants were sized and quantified by fragment analysis of fluorescently labeled PCR products using both genomic DNA where available and whole genome amplified DNA (see Supplementary Data). Mutant level was expressed as a percentage of total *PTEN* alleles. Cases with <5% total mutant were scored as wild type (WT).

### CytoSNP-850k SNP array analysis

Whole genome amplified DNA was used to determine genome-wide copy number on the Infinium CytoSNP-850k Beadchip array (Illumina, Essex, UK) according to the manufacturer's protocols. The data were analyzed using the Genotyping Module of the GenomeStudio software to calculate log R intensities and B-allele frequencies for each of the 230 single nucleotide polymorphism (SNP) markers covering the *PTEN* gene (chromosome 10: 89612850-89721667). Each sample was independently assessed for *PTEN* copy number changes by two individuals and scored as WT, heterozygous deletion or homozygous deletion.

### SNP allele quantification

Relative allele levels for the A/G SNP rs1903858 in intron 1–2 and the T/G SNP rs555895 in intron 8–9 were quantified using allele-specific restriction enzyme digestion and fragment analysis of fluorescently labeled PCR products (see Supplementary Data). A normal range using samples from 20 SNP-positive hematologically normal controls was first established; the mean allele A% for rs1903858 was 53±2% (range, 50–58%), and the mean allele T% for rs555895 was 53±2% (range, 49–57%). Any deviation from this range was considered to indicate loss of genomic material.

### Type 1 microdeletions

To screen for type 1 microdeletions as reported by Mendes *et al.*,^23^ PCR was performed with primers in introns 1–2 and 3–4 just outside of the breakpoint junction (see Supplementary Data). Resulting products were sequenced.

### Cytogenetic and fluorescence *in situ* hybridization analysis

Cytogenetic and fluorescence *in situ* hybridization analyses were performed as previously described.^7^

### Statistical analysis

Kaplan–Meier curves were used to assess overall survival (OS; the time from randomization to death), event-free survival (the time to relapse, secondary tumor or death, whichever came first) and relapse-free survival (the time to relapse or ALL death in those who achieved remission). Patients who did not experience an event were censored at the date last seen. Differences between groups were compared using the log-rank test. All *P*-values quoted are two-sided. Analyses were performed using Stata version 12.1 (Stata Corp, College Station, TX, USA).

## Results

### *PTEN* mutation analysis

#### Mutation screening

**Figure 1:**
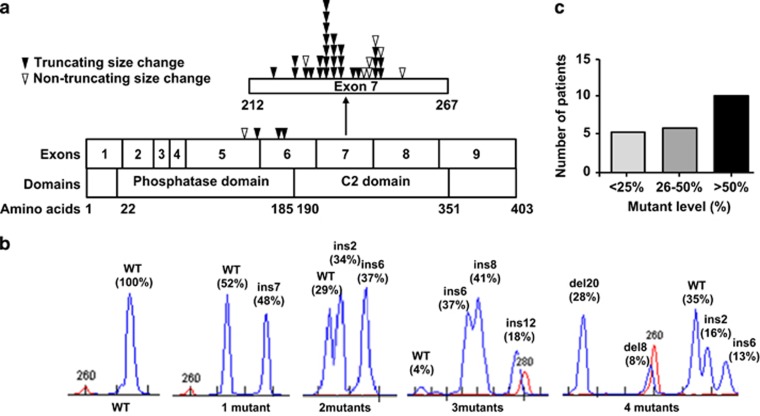
Characteristics of *PTEN* mutations detected. (**a**) Schematic representation of the location and type of mutations detected for the 39 mutants with available sequence. (**b**) Quantification of mutant level by fragment analysis, showing cases with 1–4 mutants. The level is given as a percentage of total alleles. (**c)** Distribution of total *PTEN* mutant level detected in mutant-positive patients. Del, deletion; ins, insertion.

A total of 44 *PTEN* mutations were identified in 21 of the 145 patients investigated (14%, *PTEN*^MUT^), 40 in exon 7, 2 in exon 6 and 2 in exon 5 (Supplementary Table 1). Nineteen cases (90%) had exon 7 mutations, two of which also had exon 6 mutations, and two (10%) had just exon 5 mutations (Figure 1a). The mutations included insertions, deletions and indels, with overall size changes ranging between 1 and 20 bps. In 34 (77%), the size change would cause a frameshift, and 10 (23%) were in-frame. Of the 39 with available sequence, 31 (79%) led to truncation of the C-terminus through a nonsense mutation or introduction of a premature stop codon, 8 (21%) were in-frame and non-truncating. Of note, of the 21 mutated cases, only 7 (33%) had a single mutant; 8 (38%) had two mutants, and 3 cases (14%) each had three or four mutants (Figure 1b).

#### Mutant level quantification

Exon 7 mutant levels were quantified using both genomic DNA and whole genome amplified DNA; highly comparable results were obtained (Supplementary Figure 2). The mean mutant level for individual mutants was 20% (range, 2–48%). For the exon 5 and 6 mutants, an estimated mutant level was determined from the sequence peak heights. The median total mutant level for the 21 *PTEN*^MUT^ cases was 48% (range, 10–96%); 5 cases (24%) had ⩽25% total mutant, 6 (29%) had 26–50% and 10 (47%) had >50% (Figure 1c). Where multiple mutations were detected, it was not possible to determine whether these mutants were in the same or different cell populations.

### *PTEN* copy number analysis

#### SNP arrays

Interpretable results were obtained from Illumina CytoSNP-850k SNP array analysis of 139 samples. Genomic loss of the *PTEN* gene was detected in 14 patients (10%) and scored as heterozygous loss in 11 cases and homozygous loss in 3 cases (Supplementary Figure 3). A further four cases had evidence of amplification of the *PTEN* gene, but as they did not demonstrate loss-of-function and the consequence of the amplification was not known, they were scored as WT for the purposes of this study. In all cases, the deletion looked to encompass the whole gene.

#### SNP allele quantification

**Figure 2:**
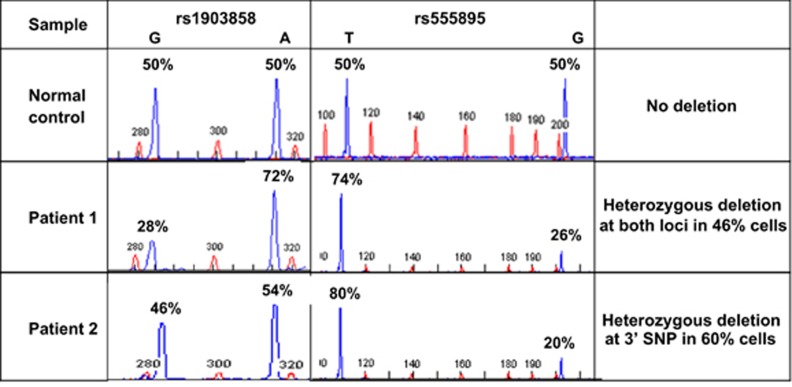
Representative SNP allele quantification at loci in *PTEN* intron 1–2 (rs1903858) and intron 8–9 (rs555895) in samples from a normal control and two patients with heterozygous loss.

In 72 of the 145 samples (50%), it was possible to validate the array data and quantify the level of heterozygous loss by measuring the relative allele levels of two common intronic SNPs in introns 1–2 (rs1903858) and 8–9 (rs555895) (Figure 2). Consistent SNP array and SNP allele quantification results were obtained in 70 cases (97%), including 64 WT, 3 with amplification and 3 with heterozygous deletion. However, in two cases that were initially scored as WT on the array, SNP allele quantification indicated loss at the 3' end of the gene. The deletions were confirmed by re-evaluation of the array data. SNP allele quantification data were also obtained on four cases without array data, three were WT and one had a heterozygous deletion. Of the six quantifiable cases with genomic loss, the SNP allele ratios were consistent with heterozygous deletion in at least half of the cells (range, 46–94%) (Figure 2, Supplementary Table 1).

#### Type 1 microdeletions

Type 1 microdeletions, removing a 65 kb region encompassing exons 2 and 3,^21^ were detected in four of the 145 patients (3%) and had not been detected by the SNP array. However, all four patients harbored other *PTEN* abnormalities. One had a homozygous deletion, one a heterozygous deletion and two had mutations with <20% total mutant level (Supplementary Table 1). Although it was not possible to determine the level of the microdeletions, in each case, the intensity of the PCR product was more likely to indicate their presence in minor sub-clones.

### Combined *PTEN* genotype

Overall, 143 patients had *PTEN* mutation and deletion status; deletion status was not available for the two remaining patients but both were *PTEN*^MUT^. In total, therefore, 32 patients (22%) had *PTEN* abnormalities (*PTEN*^ABN^), comprising 17 *PTEN*^MUT^ (53% of *PTEN*^ABN^ cases), 11 (34%) with genomic deletion (*PTEN*^DEL^) and 4 (13%) with both mutation and deletion (*PTEN*^MUT+DEL^). Of the 21 *PTEN*^MUT^ cases, 11 were considered to harbor monoallelic mutations (*PTEN*^MONO^) as the total mutant level was <50% of total alleles and there was either a single heterozygous clone (one mutant, level 37–48%, that is, present in most cells) or multiple sub-clones (more than one mutant, individual level 2–21%) (Supplementary Table 1). The remaining 10 *PTEN*^MUT^ patients were considered to harbor biallelic mutations (*PTEN*^BI^) as all had >50% total mutant consistent with either a single homozygous or hemizygous mutation or compound heterozygous mutations in at least some of the cells. Therefore, for the combined *PTEN*^MUT^ and *PTEN*^DEL^ genotype, 19 patients (59%) were scored as *PTEN*^MONO^ and 13 patients (41%) as *PTEN*^BI^.

### *RAS* mutation screening

Missense *RAS* mutations (*RAS*^MUT^) were detected in 13 (9%) of the 145 patients analyzed, 8 in *N-RAS* (4 G12D, 1 each G12V, G13C, G13R and Y64N) and 5 in *K-RAS* (2 G12C, 1 each G12S, A18D and Y64D). Only 1 of the 32 *PTEN*^ABN^ patients (3%) had a *RAS* mutation. Therefore, 44 patients (30%) were *PTEN*/*RAS*^ABN^.

### Characteristics of T-ALL patients according to *PTEN* and *RAS* genotype

There was no significant difference in sex, white blood cell count, age group, NCI risk group or cytogenetic characteristics between *PTEN*^ABN^ and *PTEN*^WT^ patients (Table 1). However, *PTEN*^ABN^ patients had a significantly higher incidence of CNS disease than *PTEN*^WT^ patients (*P*=0.04). No significant differences were observed according to *RAS* genotype.

### Prognostic relevance of *PTEN* abnormalities and *RAS* mutations

#### Response to therapy

Response to therapy data was available for all patients, and the incidence of a slow early response did not statistically differ according to the *PTEN*, *RAS* or combined *PTEN/RAS* genotype (Table 2). MRD status at day 29 post diagnosis was available for 134 patients, and no statistically significant differences were seen in the frequency of unfavorable disease according to the different genotype groups (Table 2). When the data were stratified according to the level of *PTEN* abnormality, there was a suggestion that patients with *PTEN*^MONO^ had the highest levels of MRD, but the subgroups were small and this could have occurred by chance.

#### Long-term outcome

**Figure 3:**
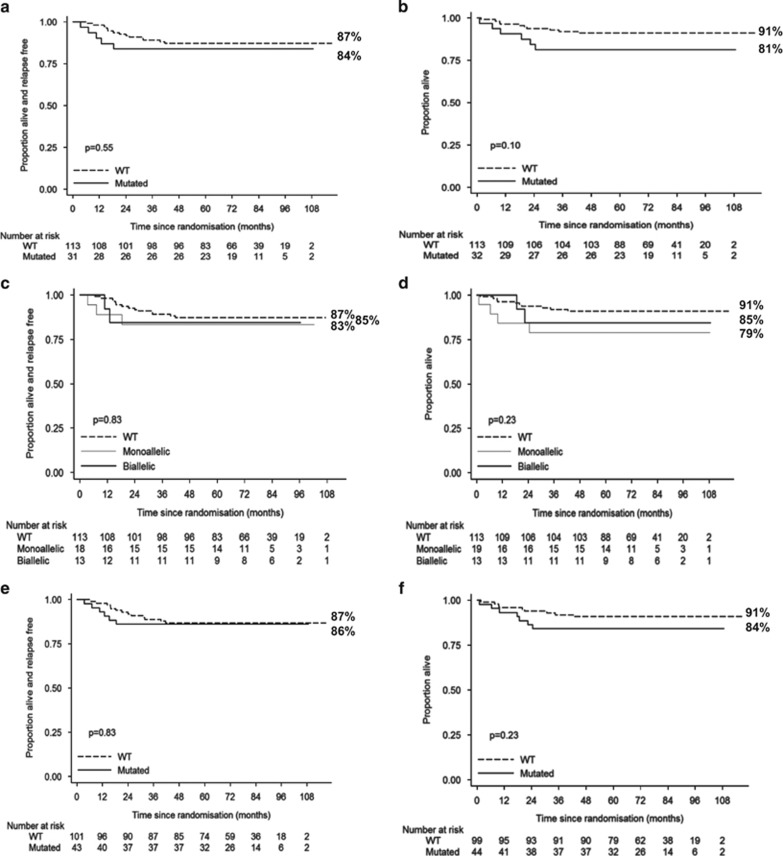
Kaplan–Meier curves for relapse-free survival (**a**, **c**, **e**) and overall survival (**b**, **d**, **f**) stratified according to *PTEN* and *RAS* genotype. (**a**, **b**) *PTEN*^ABN^ and *PTEN*^WT^. (**c**, **d**) *PTEN*^WT^, *PTEN*^MONO^ and *PTEN*^BI^. (**e**, **f**) *PTEN/RAS*^ABN^ and *PTEN/RAS*^WT^. ABN, abnormal; BI, biallelic; MONO, monoallelic.

Event-free survival, relapse-free survival and OS were slightly worse for *PTEN*^ABN^ compared with *PTEN*^WT^ cases (Figures 3a and b) (Table 2). However, the difference only showed a borderline trend for significance for OS (81 versus 91% at 5 years; hazard ratio (95% confidence interval), 2.27 (0.82–6.24); *P*=0.10), there were only a small number of events, and the confidence intervals for all analyses were wide. There was no difference in the relative proportion of *PTEN*^ABN^ and *PTEN*^WT^ patients treated on the different regimens (Table 1). When patients were stratified according to *PTEN* mutant level, *PTEN*^MONO^ had the lowest event-free survival and OS, but the number of patients in these groups was small and the differences were not significant across the three groups (Table 2) (Figures 3c and d). Similarly, outcome did not differ in the *RAS* genotypic groups (Supplementary Figures 4A and B), or in the combined *PTEN/RAS* groups (Figures 3e and f).

### *NOTCH1*/*FBXW7*/*PTEN*/*RAS* genotype of T-ALL patients

We have previously shown that outcome in this cohort differs according to the *NOTCH1*/*FBXW7* genotype, with a very good outcome in the *NOTCH1*±*FBXW7*^Double^ group.^8^ We therefore examined whether *PTEN* or *RAS* genotype changed this. The incidence of *PTEN* abnormalities did not differ according to *NOTCH1*/*FBXW7* genotype (59% *PTEN*^ABN^ patients had a *NOTCH1* and/or *FBXW7* mutation compared with 68% *PTEN*^WT^ patients; *P*=0.35), nor did *RAS* genotype (77% for *RAS*^MUT^ versus 65% for *RAS*^WT^; *P*=0.54) or the combined *PTEN*/*RAS* genotype (59% for *PTEN/RAS*^ABN^ versus 65% for *PTEN/RAS*^WT^; *P*=0.67). There were also no differences in *PTEN* and/or *RAS* genotype when the patients were grouped into the three previously defined *NOTCH1*/*FBXW7* genotype groups, *NOTCH1*^WT^*FBXW7*^WT^, *NOTCH1*^Single^*FBXW7*^WT^ and *NOTCH1*±*FBXW7*^Double^, excluding the four patients with an *FBXW7* mutation only (Supplementary Table 2).

Of the 37 *NOTCH1*±*FBXW7*^Double^ patients, 9 (24%) had *PTEN* and/or *RAS* abnormalities (5 *PTEN*^ABN^ and 5 *RAS*^MUT^, 1 with both), but this did not impact on their favorable outcome, as all were alive at 5 years (Table 3). Only two patients relapsed, both in the *PTEN/RAS*^WT^ group. In the *NOTCH1*^Single^*FBXW7*^WT^ group, 17 of the 55 patients (31%) were *PTEN/RAS*^ABN^ (14 *PTEN*^ABN^, 3 *RAS*^MUT^), and although OS was slightly lower than in the *PTEN/RAS*^WT^ patients, none of the differences were significant (Table 3). Overall, OS and relapse-free survival were lowest for *PTEN/RAS*^ABN^ patients in the *NOTCH1*^WT^*FBXW7*^WT^ group (16 of 49 patients (33%); 13 *PTEN*^ABN^, 3 *RAS*^MUT^), but did not significantly differ from *PTEN/RAS*^WT^ patients in this group (Figure 3f).

In a study of adult cases, a low-risk group, defined as *NOTCH1*-mutant and/or *FBXW7*-mutant without a *PTEN* or *RAS* mutation, had a significantly better outcome than the high-risk group of all other cases combined together.^14^ Using this stratification system, there was no significant difference in relapse-free survival in our cohort (87% for both), although there was a slightly improved OS in the low-risk group (94 versus 84% *P*=0.06) (Supplementary Figures 5A and B).

## Discussion

PTEN is a tumor-suppressor gene that is frequently inactivated in a wide variety of cancers leading to hyperactivation of the PI3K/AKT signaling pathway.^30^ It has an important role in the proliferation and survival of T-cell progenitors,^15^ and its loss may sustain leukemic T-cell viability in T-ALL.^31^ Furthermore, studies have associated *PTEN* genetic abnormalities with glucocorticoid resistance, one of the main causes of relapse in T-ALL, and sensitivity can be re-instated by inhibiting PI3K or AKT.^32, 33^ Knowledge of *PTEN* genotype may therefore help to refine current risk stratification strategies that are based on response to therapy, and inform clinical decisions. The available information to date suggests that *PTEN* abnormalities appear to be associated with adverse outcome in T-ALL, but the data are limited; the cohorts in some studies are small, not all studies have examined both mutations and deletions, and most studies have not considered the impact of the mutant level, in particular whether those with complete (biallelic) PTEN loss differ from those with partial (monoallelic) loss. In addition, contradictory results have been reported in pediatric and adult patients for the impact of the abnormalities in the *NOTCH1/FBXW7*-mutated subgroup,^13, 14^ which is important for clinical application as at least some studies have shown that patients with *NOTCH1/FBXW7* mutations do particularly well.^6, 7, 8^ There is also no information on the impact of *RAS* mutations in pediatric T-ALL, although in adult patients, they were associated with worse outcome and were included as poor risk in an oncogenetic classifier strategy.^14^

In the present study, inactivating *PTEN* mutations were detected in 14% of the cohort and gene deletions in 10%, with an overall incidence of 22% with *PTEN* gene abnormalities, which is in keeping with the combined data from other pediatric studies of 16% with mutations,^13, 18, 20, 23^ 6% with deletions,^18, 19, 23^ and 20% for mutations and/or deletions.^18, 23^ In common with the other studies, the mutations detected were predominantly located in exon 7 and were truncating mutations predicted to lead to haploinsufficiency, rather than the dominant-negative missense mutations that often occur in other types of cancer and that were associated with a more complete loss of PTEN activity and accelerated tumorigenesis in a mouse model.^34^ We have also screened for type 1 microdeletions as reported by Mendes *et al.*,^23^ but they were only detected in 3% of the patients and did not change the *PTEN* classification as all four positive cases had other *PTEN* abnormalities. These results indicate that to evaluate PTEN loss, both mechanisms of inactivation (mutation and deletion) need to be considered, and they confirm that *PTEN* abnormalities are likely to be a contributory factor in the pathogenesis of a significant proportion of patients with T-ALL.

A striking feature of our study was the high proportion of mutant-positive patients (67%) with more than one *PTEN* mutation. In some cases, this may reflect compound heterozygosity leading to complete absence of PTEN in a cell, but approximately half of the cases were likely to have multiple minor subclones. Similarly, the deletions in five of the six quantifiable cases (83%) were not present in all cells, and the frequency of gene deletions detected is probably under-estimated as the methodologies used were not sufficiently sensitive to detect low-level deletions. This is in keeping with the intratumoral heterogeneity now being revealed by deep-sequencing studies^35^ and suggests that acquisition of a *PTEN* mutation or deletion is more likely to be a later event associated with disease progression. It will therefore be important to determine whether these *PTEN*^ABN^ clones are being selected for at relapse. Analysis of paired diagnostic and relapse samples has demonstrated acquisition of *PTEN* mutations and deletions at relapse,^16, 36^ but whether these are *de novo* changes or therapy-induced selection is unclear. Back-tracking has shown that minor *PTEN*^ABN^ clones can be clonally expanded to become the dominant clone at relapse.^3, 36^ Xenotransplantation studies have not consistently demonstrated that loss of PTEN constitutes an adequate selection pressure for driving this expansion,^23, 36^ although in this setting, primary leukemic cells with PTEN knocked down by short hairpin RNAs did show a competitive engraftment advantage over the unmodified cells.^36^ The significance of specific abnormalities may also be highly dependent on the presence of other co-incident genetic events, and further work will be required to determine whether this can be predicted from the genetic profile of individual cases.

Despite the data implicating the PI3K/AKT pathway as an important oncogenic driver pathway in T-ALL, in pediatric patients, there is currently little evidence to suggest that the *PTEN* genotype is a strong prognostic factor. We found no significant association between *PTEN* abnormalities and either response to therapy or long-term outcome in our cohort, and this absence of an impact could not be attributed to therapy received as the proportion of patients with/without *PTEN* abnormalities treated on the different regimens did not differ, although it must be acknowledged that the number of events is small and larger studies may reveal more minor effects. There was a suggestion in our study that *PTEN* abnormalities may be associated with a worse OS and this is in general agreement with other pediatric studies.^12, 13, 20^ A meta-analysis would be useful, but few studies have reported both *PTEN* mutational and copy number status. Adding patients with *RAS* mutations to the abnormal group made no difference. Studies have suggested that the monoallelic loss of *PTEN* may be more detrimental than biallelic loss, leading to tumor progression rather than *Pten*-loss-induced cellular senescence.^37^ However, when our patients were stratified according to the level of mutant or deletion, although *PTEN*^MONO^ patients were associated with unfavorable levels of MRD and a worse OS, the difference was not significant and, in view of the small number of patients in the subgroups, this would require validation in a larger cohort.

Importantly, we found no evidence that the presence of a *PTEN* abnormality impacted on the highly favorable outcome that we previously reported in *NOTCH1*±*FBXW7*^Double^ patients.^8^ Of note, these results are in keeping with those from the pediatric ALL-BFM 2000 trial,^13^ but in distinct contrast to adult patients where a significantly adverse impact of *PTEN/RAS* abnormalities was found, irrespective of *NOTCH1/FBXW7* genotype.^14^ Applying the *NOTCH1*/*FBXW7*/*RAS*/*PTEN*-based oncogenetic classification criteria proposed for adults to our pediatric cohort, which does not discriminate between *NOTCH1*/*FBXW7* double- and single-mutated patients,^14^ there was no difference in relapse rate between the high-risk and low-risk groups and only a borderline difference in OS, which could be attributed to better salvage of relapsed patients in the low-risk group. This difference (94 versus 84%) was small compared with the major difference seen in adult patients (82 versus 44%) and with the small number of events involved, it could have occurred by chance. Larger studies will be needed to determine whether this classification system is informative in pediatric patients.

These results would therefore suggest that, at present, despite *PTEN* loss being common in pediatric T-ALL, screening for *PTEN* abnormalities at diagnosis would not add further information to refine the currently available risk-adapted therapy strategies. However, with the current development and clinical evaluation of many PI3K inhibitors,^38^ the *PTEN* genotype may serve as a potential biomarker for response to these agents.

## Figures and Tables

**Table 1:** Characteristics of cohort studied according to *PTEN* and *RAS* genotype

*Subgroup*	*Total*	*PTEN genotype*			*RAS genotype*		
		*PTEN*^*WT*^(n=*113)*	*PTEN*^*ABN*^(n=*32)*	P^a^	*RAS*^*WT*^(n=*132)*	*RAS*^*MUT*^(n=*13)*	P^a^
		No. (%)^b^	No. (%)^b^		No. (%)^b^	No. (%)^b^	
Gender				0.48			0.3
Male	111	85 (75%)	26 (81%)		99 (75%)	12 (92%)	
Female	34	28 (25%)	6 (19%)		33 (25%)	1 (8%)	
WBC (x10^9^/l)				0.15			0.79
<50	47	41 (36%)	6 (19%)		42 (32%)	5 (38%)	
50–99	22	17 (15%)	5 (16%)		21 (16%)	1 (8%)	
⩾100	76	55 (49%)	21 (66%)		69 (52%)	7 (54%)	
Median count	110.5	95	135.7		108.25	167.1	
Range	0.5–881.0	0.5–881.0	0.7–777		0.7–881.0	0.5–456.5	
Age group (years)				0.1			0.5
<10	79	56 (50%)	23 (72%)		72 (55%)	7 (54%)	
15-Oct	47	40 (35%)	7 (22%)		44 (33%)	3 (23%)	
⩾16	19	17 (15%)	2 (6%)		16 (12%)	3(23%)	
Median	9	10	8.5		9	9	
Range	1–23	1–23	1–18		1–23	4–23	
CNS disease				0.04			>0.99
No	135	110 (97%)	28 (88%)		125 (95%)	13 (100%)	
Yes	7	3 (3%)	4 (12%)		7 (5%)	0	
NCI risk group				0.99			0.67
Low	18	14 (12%)	4 (13%)		16 (12%)	2 (15%)	
High	127	99 (88%)	28 (88%)		116 (88%)	11 (85%)	
Treatment regimen (risk)				0.87			0.23
A (low/standard)	13	10 (9%)	3 (9%)		11 (8%)	2 (15%)	
B (intermediate)	83	66 (58%)	17 (53%)		78 (59%)	5 (38%)	
C (high)	49	37 (33%)	12 (38%)		43 (33%)	6 (46%)	
							
Cytogenetics				0.86c,^d^			>0.99^d^
Normal	28	21 (19%)	7 (22%)		26 (20%)	2 (15%)	
Abnormal	90	69 (61%)	21 (66%)		81 (61%)	9 (69%)	
Failed/missing	27	23 (20%)	4 (13%)		25 (19%)	2 (15%)	
Genetic subgroup				N/A			N/A
AF10-CALM	3	3 (3%)	0		2 (2%)	1 (8%)	
LMO2	10	8 (7%)	2 (6%)		9 (7%)	1 (8%)	
MLL	3	3 (3%)	0		3 (2%)	0	
TAL1	14	8 (7%)	6 (19%)		14 (11%)	0	
TLX1	5	4 (4%)	1 (3%)		5 (4%)	0	
TLX3	15	14 (12%)	1 (3%)		11 (8%)	4 (31%)	
Other/unclassified	95	73 (65%)	22 (69%)		88 (67%)	7 (54%)	
CDKN2A/B deletion				0.29			0.2
No	45	36 (32%)	9 (28%)		39 (30%)	6 (46%)	
Yes	79	56 (50%)	23 (72%)		74 (56%)	5 (38%)	
Missing	21	21 (19%)	0		19 (14%)	2 (15%)	

Abbreviations: ABN, abnormal; CNS, central nervous system; NCI, National Cancer Institute; N/A, not applicable; MUT, mutant; WT, wild type; WBC, white blood cell count.

a *P*-values: Fisher's exact test unless otherwise indicated.

b Percentages may not add up to 100 owing to rounding up.

c Chi squared test.

d Excludes missing/failed.

**Table 2:** Response to chemotherapy and survival status according to *PTEN/RAS* genotype

		*Overall PTEN genotype*			*PTEN WT/monoallelic/biallelic*				*Overall RAS genotype*			*PTEN/RAS genotype*		
	*No.*	*PTEN*^*WT*^(n=*113)*	*PTEN*^*ABN*^(n=*32)*	P^a^	*PTEN*^*WT*^(n=*113)*	*PTEN*^*MONO*^(n=*19)*	*PTEN*^*BI*^(n=*13)*	Pa,^b^	*RAS*^*WT*^(n=*132)*	*RAS*^*MUT*^(n=*13)*	P^a^	*PTEN/RAS*^*WT*^(n=*101)*	*PTEN/RAS*^*ABN*^(n=*44)*	P^a^
*SER*														
No	108	84 (74%)	24 (75%)	0.94	84 (74%)	14 (74%)	10 (70%)	>0.99^c^	100 (76%)	8 (62%)	0.32^c^	77 (76%)	31 (70%)	0.46
Yes	37	29 (26%)	8 (25%)		29 (26%)	5 (26%)	3 (30%)		32 (24%)	5 (38%)		24 (24%)	13 (30%)	
														
*MRD at day 29*														
Negative	72	60 (56%)	12 (44%)	0.28	60 (56%)	5 (31%)	7 (64%)	0.69^b^	66 (55%)	6 (46%)	0.56	54 (57%)	18 (46%)	0.26
Positive	62	47 (44%)	15 (56%)		47 (44%)	11 (69%)	4 (36%)		55 (45%)	7 (54%)		41 (43%)	21 (54%)	
														
*Events/*n, *5-year outcome, % (95% CI)*														
RFS	144	14/113	5/31	0.55^d^	14/113	3/18	2/13	0.60b,^d^	18/131	1/13	0.54^d^	13/101	6/43	0.83^d^
		87%	84%		87%	83%	85%		86%	92%		87%	86%	
		(80–92%)	(66–93%)		(80–92%)	(57–94%)	(51–96%)		(79–91%)	(57–99%)		(78-92%)	(72-94%)	
														
EFS	145	18/113	7/32	0.37^d^	18/113	5/19	2/13	0.61b,^d^	22/132	2/13	0.83^d^	17/101	8/44	0.77^d^
		85%	78%		85%	74%	85%		84%	85%		84%	82%	
		(77–90%)	(60–90%)		(77–90%)	(48–88%)	(51–96%)		(76–89%)	(51–96%)		(76-90%)	(67-91%)	
														
OS	145	10/113	6/32	0.10^d^	10/113	4/19	2/13	0.20b,^d^	15/132	1/13	0.69^d^	9/101	7/44	0.20^d^
		91%	81%		91%	79%	85%		89%	92%		91%	84%	
		(84–95%)	(63–91%)		(84–95%)	(53–92%)	(51–96%)		(82–93%)	(57–99%)		(84-95%)	(70-92%)	

Abbreviations: ABN, abnormal; BI, biallelic; CI, confidence interval; EFS, event-free survival; MONO, monoallelic; MRD, minimal residual disease; OS, overall survival; RFS, relapse-free survival; SER, slow early response; WT, wild type.

a *P*-values: Chi-squared test unless otherwise indicated.

b Test for trend across *PTEN*^WT^, *PTEN*^MONO^ and *PTEN*^BI^ groups.

c Fisher's exact test.

d Log-rank test.

**Table 3:** Outcome at 5 years according to *PTEN/RAS* genotype in the *NOTCH1/FBXW7* subgroups

*NOTCH1/FBXW7 subgroup*	*PTEN/RAS genotype*	*RFS*			*OS*		
		*Events/*n	*% (95% CI)*	P^a^	*Events/*n	*% (95% CI)*	P^a^
*NOTCH1*±*FBXW7*^Double^	*PTEN*^WT^	2/32	94% (77–98%)	0.57	0/32	100%	N/A^b^
	*PTEN*^ABN^	0/5	100%		0/5	100%	
	*PTEN/RAS*^WT^	2/28	93% (74–98%)	0.42	0/28	100%	N/A^b^
	*PTEN/RAS*^ABN^	0/9	100%		0/9	100%	
							
*NOTCH1*^Single^*FBXW7*^WT^	*PTEN*^WT^	7/41	83% (67–93%)	0.68	4/41	90% (76–96%)	0.24
	*PTEN*^ABN^	3/14	79% (47–93%)		3/14	79% (47–93%)	
	*PTEN/RAS*^WT^	7/38	81% (64–91%)	0.98	4/38	89% (74–96%)	0.44
	*PTEN/RAS*^ABN^	3/17	82% (55–94%)		3/17	82% (55–94%)	
							
*NOTCH1*^WT^*FBXW7*^WT^	*PTEN*^WT^	5/36	85% (68–94%)	0.82	6/36	83% (67–92%)	0.60
	*PTEN*^ABN^	3/13	83% (48–96%)		3/13	77% (47–93%)	
	*PTEN/RAS*^WT^	5/33	87% (69–95%)	0.49	5/33	85% (67–93%)	0.39
	*PTEN/RAS*^ABN^	4/16	80% (50–93%)		4/16	75% (46–90%)	

Abbreviations: ABN, abnormal; CI, confidence interval; OS, overall survival; RFS, relapse-free survival; WT, wild type.

a *P*-values: Log-rank test.

b Not applicable as no deaths reported.
