# Supplementary material for: Impact of PTEN abnormalities on outcome in pediatric patients with T-cell acute lymphoblastic leukemia treated on the MRC UKALL2003 trial
Source: Leukemia. 2015 Aug 21;30(1):39–47. doi: 10.1038/leu.2015.206 (PMC4705426; doi:10.1038/leu.2015.206)
Supplement: Supplementary Figures [file leu2015206x1.ppt]

## Slide 1
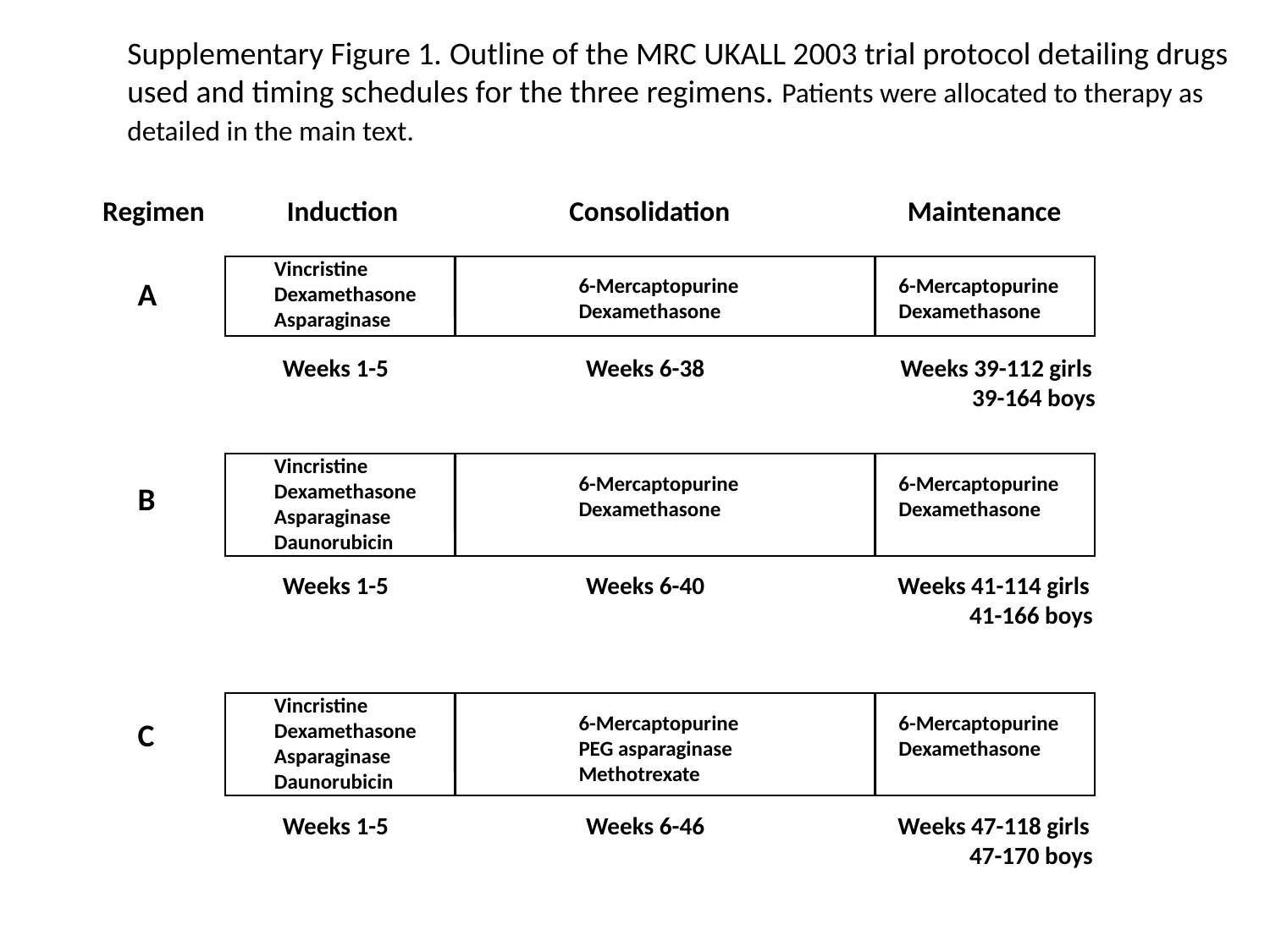

Supplementary Figure 1. Outline of the MRC UKALL 2003 trial protocol detailing drugs used and timing schedules for the three regimens. Patients were allocated to therapy as detailed in the main text.
Regimen Induction Consolidation Maintenance
Vincristine
Dexamethasone
Asparaginase
6-Mercaptopurine
Dexamethasone
6-Mercaptopurine
Dexamethasone
A
Weeks 1-5
Weeks 6-38
Weeks 39-112 girls
 39-164 boys
Vincristine
Dexamethasone
Asparaginase
Daunorubicin
6-Mercaptopurine
Dexamethasone
6-Mercaptopurine
Dexamethasone
B
Weeks 1-5
Weeks 6-40
Weeks 41-114 girls
 41-166 boys
Vincristine
Dexamethasone
Asparaginase
Daunorubicin
6-Mercaptopurine
PEG asparaginase
Methotrexate
6-Mercaptopurine
Dexamethasone
C
Weeks 1-5
Weeks 6-46
Weeks 47-118 girls
 47-170 boys

## Slide 2
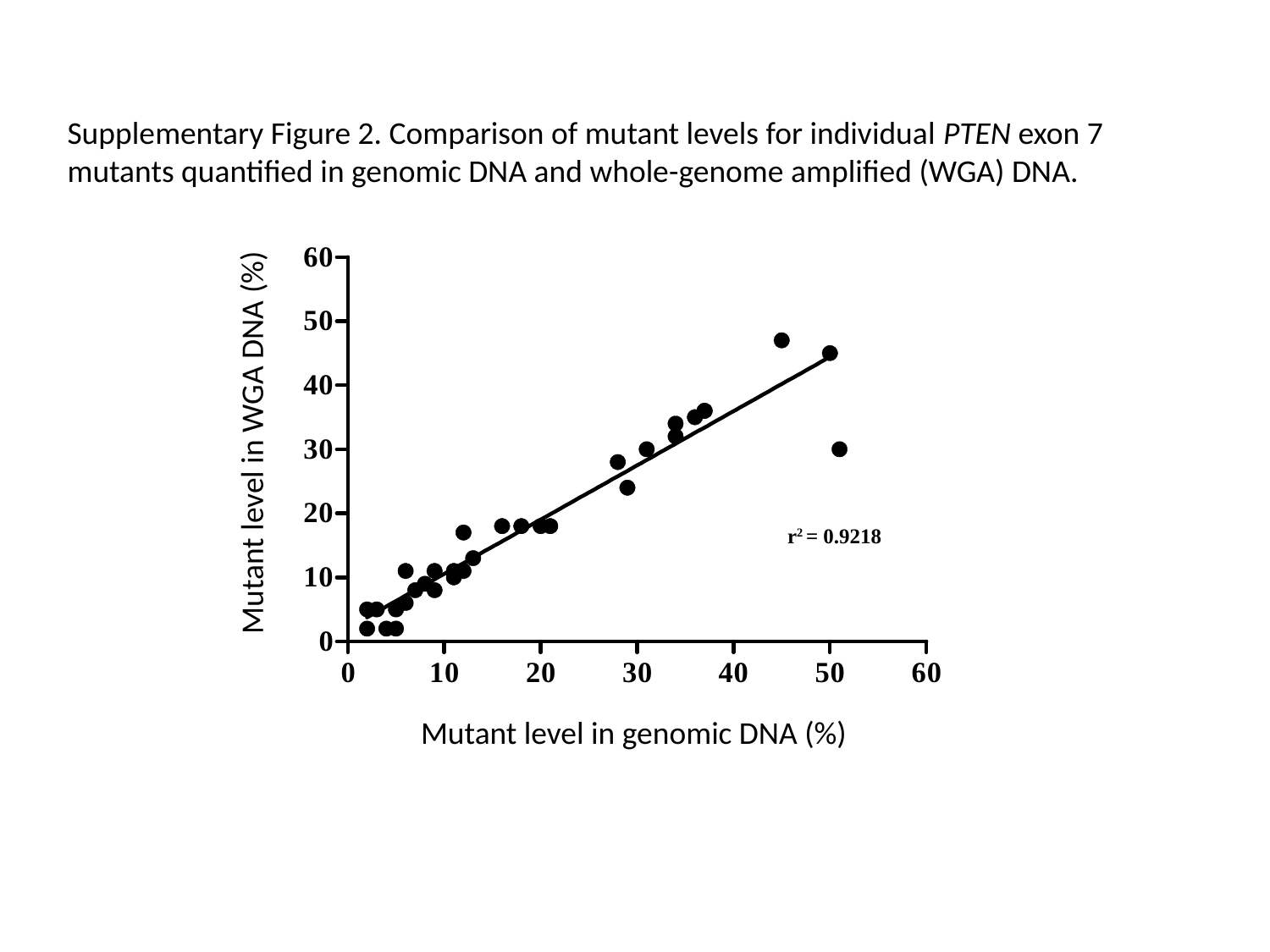

Supplementary Figure 2. Comparison of mutant levels for individual PTEN exon 7 mutants quantified in genomic DNA and whole-genome amplified (WGA) DNA.
r2 = 0.9218
Mutant level in WGA DNA (%)
Mutant level in genomic DNA (%)

## Slide 3
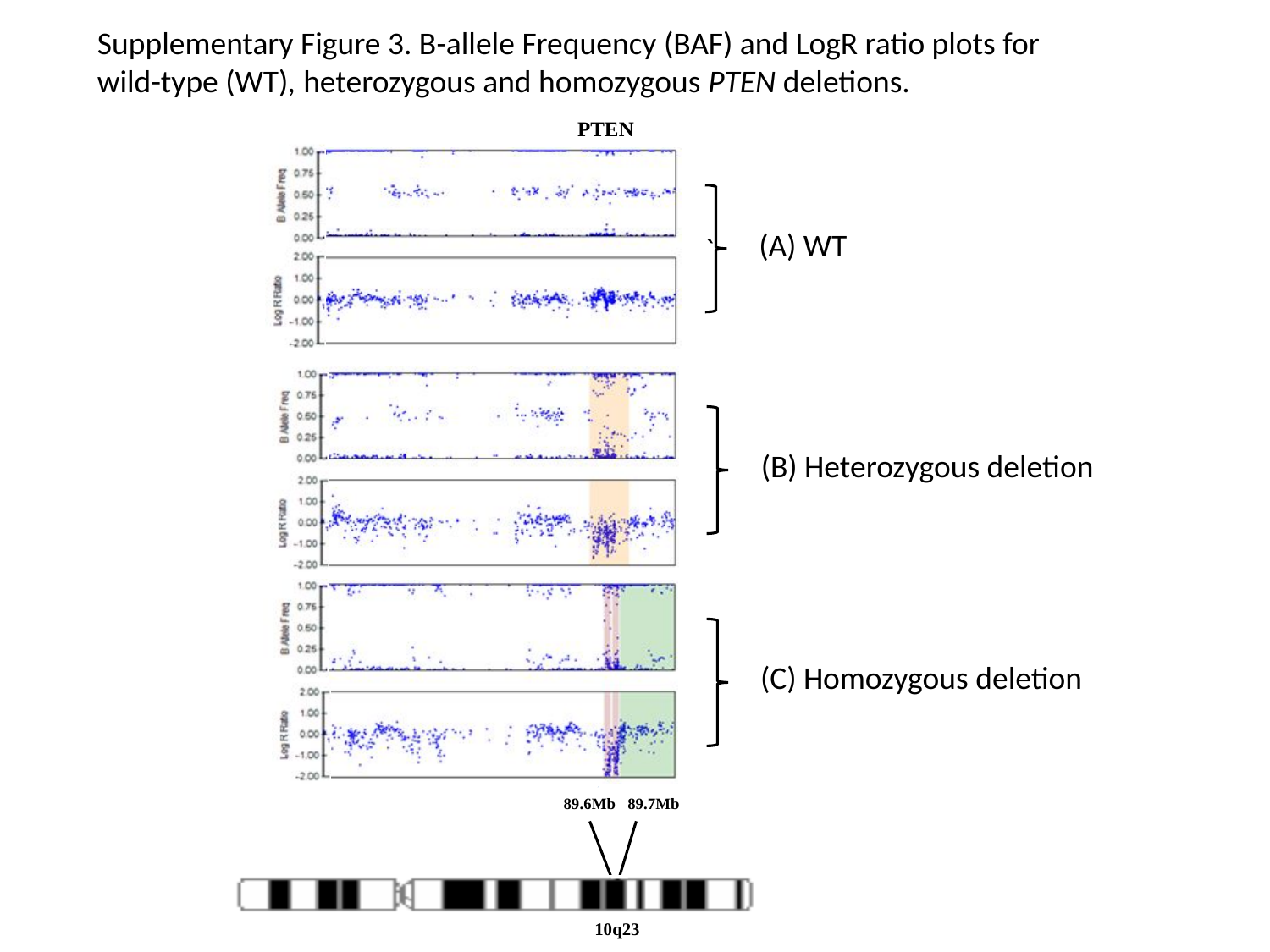

Supplementary Figure 3. B-allele Frequency (BAF) and LogR ratio plots for wild-type (WT), heterozygous and homozygous PTEN deletions.
PTEN
89.6Mb
89.7Mb
10q23
`
(A) WT
(B) Heterozygous deletion
(C) Homozygous deletion

## Slide 4
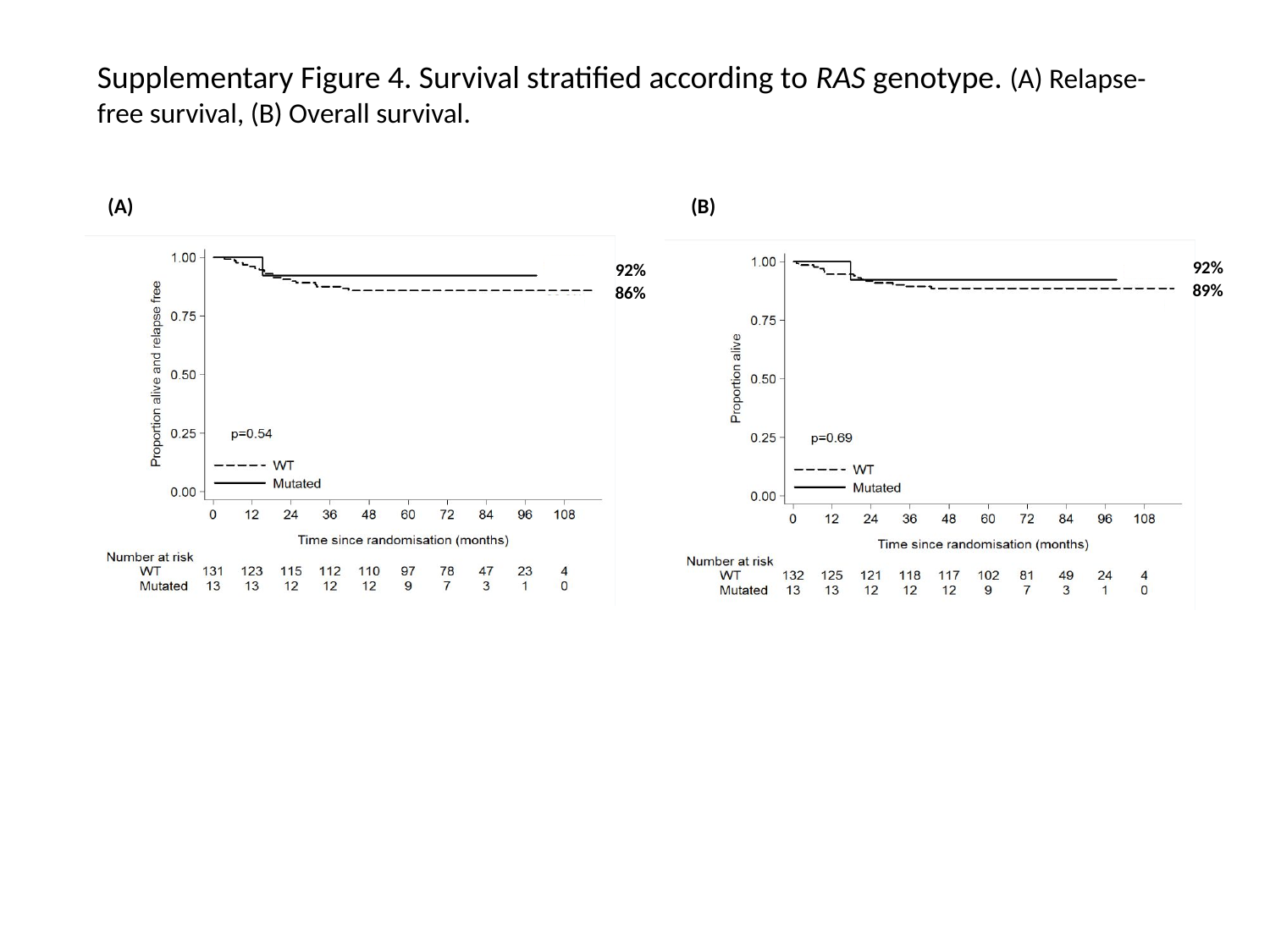

Supplementary Figure 4. Survival stratified according to RAS genotype. (A) Relapse-free survival, (B) Overall survival.
(A)
(B)
92%
92%
89%
86%

## Slide 5
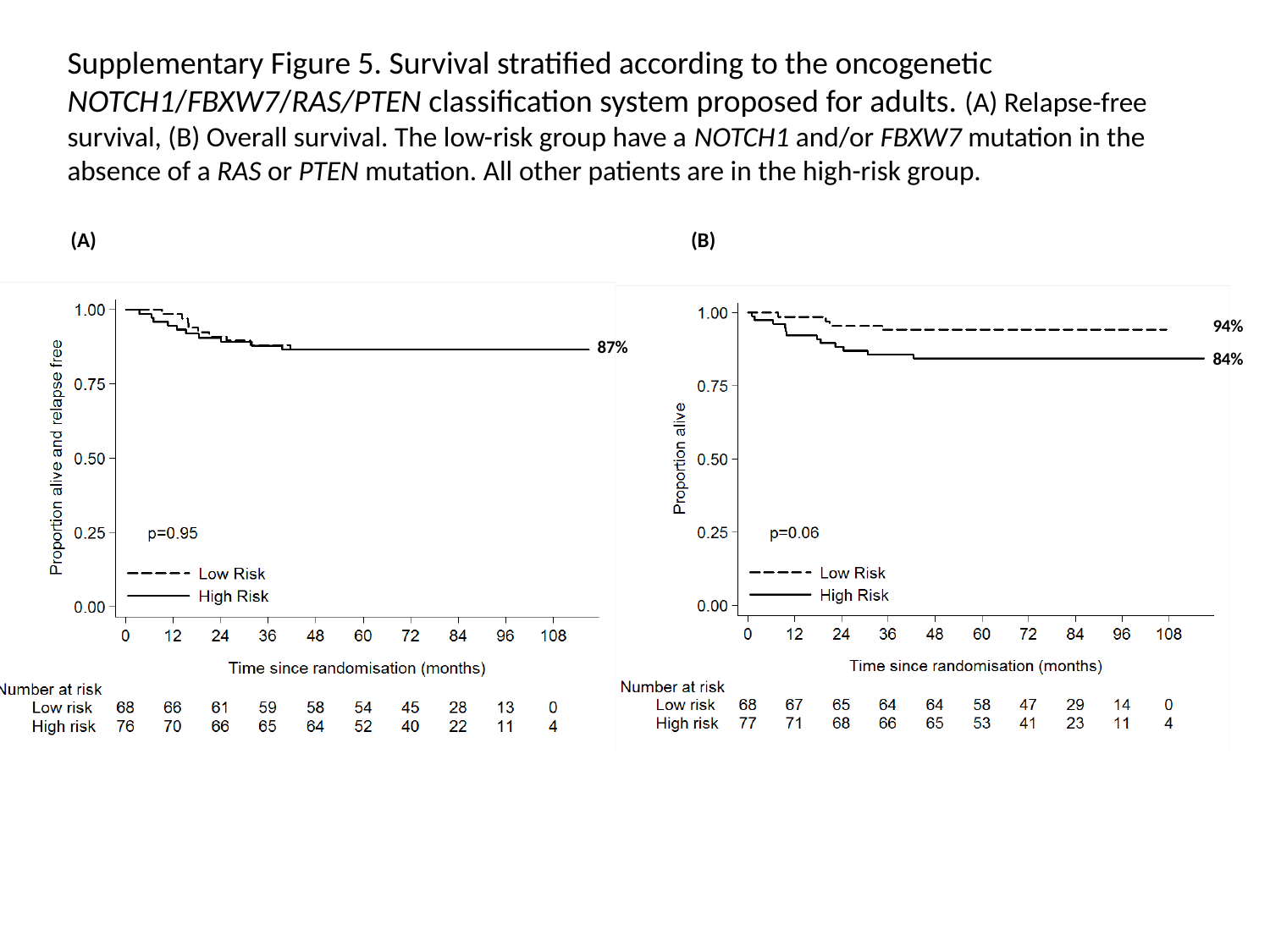

Supplementary Figure 5. Survival stratified according to the oncogenetic NOTCH1/FBXW7/RAS/PTEN classification system proposed for adults. (A) Relapse-free survival, (B) Overall survival. The low-risk group have a NOTCH1 and/or FBXW7 mutation in the absence of a RAS or PTEN mutation. All other patients are in the high-risk group.
(A)
(B)
87%
94%
84%
