# Supplementary material for: Impact of PTEN abnormalities on outcome in pediatric patients with T-cell acute lymphoblastic leukemia treated on the MRC UKALL2003 trial
Source: Leukemia. 2015 Aug 21;30(1):39–47. doi: 10.1038/leu.2015.206 (PMC4705426; doi:10.1038/leu.2015.206)
Supplement: Supplementary Information [file leu2015206x2.doc]

**DATA SUPPLEMENT**

**Mutation Screening**

Polymerase chain reaction (PCR) products for exons 1-9 of the *PTEN* gene, exons 2 and 3 of the *N-RAS* and *K-RAS* genes were amplified from genomic DNA (gDNA) using Optimase Polymerase (Transgenomic, Glasgow, UK) or Phusion High-Fidelity DNA Polymerase (New England BioLabs, Hitchin, UK) according to manufacturer’s instructions, with 35 cycles of amplification, and primers and annealing temperatures as specified in Supplemental Table 3. PCR products from the patient’s samples mixed in equal quantities with products from a known wild-type control, denatured, re-annealed slowly to allow heteroduplex formation and then analyzed on a denaturing high-performance liquid chromatography (dHPLC) WAVE platform (Transgenomic, Glasgow, UK) at optimal melting temperatures calculated using Transgenomic’s Navigator software (Supplementary Table S3). Samples with abnormal WAVE chromatograms were sequenced.

**Whole genome amplification (WGA)**

1-10ng of DNA from all samples was whole genome amplified using the REPLI-g Mini Kit (Qiagen, Crawley, UK) according to manufacturer’s instructions.

**Quantification of mutant level**

For quantification of the relative mutant level of *PTEN* exon 7 mutants, fluorescently-labeled PCR products were prepared from both gDNA and WGA-DNA using BIOTAQ DNA polymerase (BIOLINE, London UK) and the same PCR conditions as before except that the forward primer was fluorescently labeled, the primer concentration was halved, and the number of cycles was reduced to 28. The products were analyzed by fragment analysis on a CEQ8000 DNA Genetic Analysis System (Beckman Coulter UK Ltd., High Wycombe, UK). The relative mutant level was calculated using the area under the peak and expressed as a percentage of total alleles.

For one sample harboring a c.696delCinsGG mutation (p.R233fs) that could not be resolved from the WT peak in fragment analysis, the labeled PCR product was digested with 1µl *Hpy99I* (New England Biolabs, Hitchin, UK) before size separation. Mutant alleles were undigested giving a 267bp fragment and WT alleles were digested to a labeled 121bp fragment. Similarly, for another sample with a c.696insT mutation (p.R233fs), labeled PCR product was digested with *Taqα1* (New England Biolabs) before analysis. WT alleles were uncut giving a 267bp fragment and mutant alleles were digested to a labeled 115bp fragment.

**SNP allele quantification**

Cases that were heterozygous for the A/G SNP in *PTEN* intron 1-2 (rs1903858) were identified from a characteristic WAVE chromatogram obtained when screening for mutations in *PTEN* exon 2. Samples were screened for heterozygosity of the T/G SNP in *PTEN* intron 8-9 (rs555895) by dHPLC of amplicons obtained using primers Int8-9F and Int8-9R (Supplementary Table 3). For SNP-informative cases, PCR products were obtained as before except that the forward primer was fluorescently-labeled, the number of cycles was reduced to 28 and BIOTAQ DNA polymerase was used. For rs1903858, the PCR products were digested with *Hind*III (New England Biolabs) before analysis on the CEQ 8000 Genetic Analysis System. The A allele products were undigested giving a 314bp fragment and the G allele products were digested to a labeled 281bp fragment. For rs555895, the PCR products were digested with *Hinc*II (New England Biolabs) before size separation. G alleles were uncut giving a 201bp fragment and the T alleles were digested to a labeled 110bp fragment.

**Type 1 microdeletions**

Type-1 microdeletions with breakpoints in *PTEN* intron 1-2 and 3-4 were screened by agarose gel electrophoresis of PCR products obtained using 35 cycles of amplification, BIOTAQ DNA Polymerase, and primers and conditions as given in Supplementary Table 3. A PCR product of approximately 306bp or more was indicative of a deletion and was sequenced for confirmation.

Supplementary Table S1. Details of the *PTEN* abnormalities in the 32 *PTEN*^ABN^ patients

| No. | Mut/∆ | No. Mut. | Size change (% Mut) | Protein change | Total % Mut | SNP array | SNP allele quantification (Mean allele %) | | Type 1 Micro∆ | % cells deleted | *NOTCH1/FBXW7* genotype |
| --- | --- | --- | --- | --- | --- | --- | --- | --- | --- | --- | --- |
|  |  |  |  |  |  |  | rs1903858 A:G | rs555895 T:G |  |  |  |
| Biallelic | | | | | | | | | | | |
| 1 | ∆ | - |  |  |  | Hom ∆ | N/A | N/A | Present |  | Double |
| 2 | ∆ | - |  |  |  | Hom ∆ | N/A | N/A | ND |  | W |
| 3 | ∆ | - |  |  |  | Hom ∆ | N/A | N/A | ND |  | W |
| 4 | Mut | 3 | Ins8 (41%)  Ins6 (37%)  Ins12 (18%) | Y225X  P246_L247insAP  Q245_P246delinsSPLVPA | 96% | W | 53:47 | 50:50 | ND |  | W |
| 5 | Mut+∆ | 4 | Del12 (31%)  Ins13 (24%*)  Ins4 (18%)  Ins14 (3%) | N228_R232delinsI  S179fs  L247fs  N/A | 76%* | Het ∆ | N/A | N/A | ND |  | W |
| 6 | Mut | 2 | Ins1 (39%)  Ins16 (34%) | P246fs  P246fs | 73% | N/A | N/A | N/A | ND |  | Double |
| 7 | Mut+∆ | 1 | Ins1 (70%*) | V166fs | 70%* | Het ∆ | N/A | N/A | ND |  | Double |
| 8 | Mut | 2 | Ins6 (37%)  Ins2 (33%) | P244_P246delinsLPLRS  E235fs | 70% | W | 48:52 | 52:48 | ND |  | Single |
| 9 | Mut | 4 | Del20 (28%)  Ins2 (17%)  Ins6 (13%)  Del8 (9%) | Q219fs  R234fs  Q245_P246insIP  N/A | 67% | W (Amp) | N/A | N/A | ND |  | W |
| 10 | Mut | 2 | Ins4 (45%)  Ins1 (17%) | L247fs  R233fs | 62% | W | N/A | N/A | ND |  | W |
| 11 | Mut+∆ | 1 | Ins9 (60%*) | Q149_E150insRPPV | 60%* | Het ∆ | 53:47 | 80:20 | ND | 75% (3’) | Single |
| 12 | Mut | 3 | Ins8 (29%*)  Ins18 (27%)  Ins2 (4%) | K183fs  V255_E256insPQLPTS  N/A | 60%* | W | N/A | N/A | ND |  | W |
| 13 | Mut | 2 | Del20 (28%)  Ins1 (28%) | N228fs  R233fs | 56% | W | 55:45 | 47:53 | ND |  | Single |
| Monoallelic | | | | | | | | | | | |
| 14 | ∆ | - |  |  |  | Het ∆ | N/A | N/A | ND |  | W |
| 15 | ∆ | - |  |  |  | Het ∆ | N/A | N/A | ND |  | Single |
| 16 | ∆ | - |  |  |  | Het ∆ | N/A | N/A | ND |  | Single |
| 17 | ∆ | - |  |  |  | Het ∆ | N/A | N/A | ND |  | Double |
| 18 | ∆ | - |  |  |  | N/A | 73:27 | 75:25 | Present | 65% | Single |
| 19 | ∆ | - |  |  |  | Het ∆ | 17:83 | 22:78 | ND | 76% | Single |
| 20 | ∆ | - |  |  |  | Het ∆ | 7:93 | 5:95 | ND | 94% | Single |
| 21 | ∆ | - |  |  |  | Het ∆ | 53:47 | 21:79 | ND | 73% (3’) | Single |
| 22 | Mut | 1 | Ins7 (48%) | T232fs | 48% | W | 52:48 | 52:48 | ND |  | Single |
| 23 | Mut | 1 | Ins1 (47%) | E235fs | 47% | W | 51:49 | 50:50 | ND |  | W |
| 24 | Mut | 1 | Ins2 (41%) | R233fs | 41% | W | 51:49 | 54:46 | ND |  | Single |
| 25 | Mut | 1 | Ins12 (37%) | R233_E235delinsKNHX | 37% | W | 57:43 | 51:49 | ND |  | Single |
| 26 | Mut | 4 | Ins4 (15%)  Del13 (9%)  Ins7 (6%)  Ins11 (4%) | R233fs  S229fs  R233fs  T232fs | 34% | W | N/A | N/A | ND |  | W |
| 27 | Mut | 2 | Ins8 (21%)  Ins4 (6%) | R242fs  Y225fs | 27% | N/A | N/A | N/A | ND |  | Single |
| 28 | Mut | 2 | Ins5 (12%)  Ins4 (11%) | R234fs  R234fs | 23% | W | N/A | N/A | ND |  | W |
| 29 | Mut | 3 | Ins3 (9%)  Ins5 (6%)  Ins1 (2%) | L247_P248insL  R234fs  R233fs | 17% | W (Amp) | 68:32 | 68:32 | Present |  | W |
| 30 | Mut | 2 | Ins14 (11%)  Del13 (2%) | F241fs  N/A | 13% | W | 52:48 | 50:50 | ND |  | Single |
| 31 | Mut+∆ | 2 | Ins9 (9%)  Ins11 (3%) | P246LSSFX  N/A | 12% | Het ∆ | 68:32 | 62:38 | ND | 46% | W |
| 32 | Mut | 1 | Ins10 (10%) | E235fs | 10% | W | N/A | N/A | Present |  | Double |

*Mutant level estimated from sequence

Abbreviations: ∆, deletion; Del, deletion; fs, frameshift; Hom, homozygous; Het, heterozygous; Ins, insertion; Mut, Mutant; N/A, not available; ND, not detected; W, wild-type

Supplementary Table S2. *NOTCH1/FBXW7* genotype in the *PTEN* and *RAS* subgroups

| *NOTCH1/FBXW7* genotype | Total | *NOTCH1* and/or *FBXW7*^WT^ (%)  (n=49) | *NOTCH1* and/or *FBXW7*^MUT^ (%)  (n=96) | *P** |  |
| --- | --- | --- | --- | --- | --- |
| *PTEN*^WT^  *PTEN*^ABN^  *RAS*^WT^  *RAS*^MUT^  *PTEN/RAS*^WT^  *PTEN/RAS*^ABN^ | 113  32  132  13  101  44 | 36 (73%)  13 (27%)  46 (94%)  3 (6%)  33 (67%)  16 (33%) | 77 (80%)  19 (20%)  86 (90%)  10 (10%)  68 (71%)  28 (29%) | 0.35  0.54^#^  0.67 |  |
|  |  |  |  |  |  |
| *NOTCH1/FBXW7* genotype |  | *NOTCH1*^WT^*FBXW7*^WT^ (%)  (n=49) | *NOTCH1*^Single^*FBXW7*^WT†^ (%)  (n=55) | *NOTCH1±FBXW7*^Double†^ (%)  (n=37) | *P** |
| *PTEN*^WT^  *PTEN*^ABN^  *RAS*^WT^  *RAS*^MUT^  *PTEN/RAS*^WT^  *PTEN/RAS*^ABN^ | 109  32  130  11  99  42 | 36 (73%)  13 (27%)  46 (94%)  3 (6%)  33 (67%)  16 (33%) | 41 (75%)  14 (25%)  52 (95%)  3 (5%)  38 (69%)  17 (31%) | 32 (86%)  5 (14%)  32 (86%)  5 (14%)  28 (76%)  9 (24%) | 0.17^∆^  0.35^#^  0.42^∆^ |

**P* values are for Chi-squared test except where otherwise indicated. ^#^Fisher’s exact test. ^∆^Test for trend

^†^Excludes 4 *NOTCH1*^WT^*FBXW7*^MUT^ patients

Supplementary Table S3. Primer sequences and conditions for PCR and WAVE analysis

| Amplicon | Primer | Sequence | Annealing temperature (^o^C) | WAVE analysis temperature (^o^C) |
| --- | --- | --- | --- | --- |
| PTEN exon 1 | F  R | 5’-AGAGCCATTTCCATCCTGCAGA-3’  5’-AACTACGGACATTTTCGCATCCG-3’ | 63 | 59.6 |
| PTEN exon 2 | F  R | 5’-CACCTTTTATTACTGCAGCTAT-3’  5’-CACAAAGTATCTTTTTCTGTGG-3’ | 57 | 54.1 |
| PTEN exon 3 | F  R | 5’-CAAATGTTAGCTCATTTTTGTT-3’  5’-GTTAAAATGTATCTTAACTCT-3’ | 51 | 54.7 |
| PTEN exon 4 | F  R | 5’GTACTTTTTTTTCTTCCTAAGTGCAAAAG-3’  5’-TCACTCGATAATCTGGATGACTCA-3’ | 62 | 56 |
| PTEN exon 5 | F  R | 5’-GAGTTTTTTTTTCTTATTCTGAGGTTATC-3’  5’-CTCAGATCCAGGAAGAGGAAAG-3’ | 62 | 55.5, 57.2 |
| PTEN exon 6 | F  R | 5’-GGCTACGACCCAGTTACCATAG-3’  5’-CTTCTAGATATGGTTAAGAAAACTGTTC-3’ | 62 | 57.1 |
| PTEN exon 7 | F  R | 5’-GACAGTTAAAGGCATTTCCTG-3’  5’-GTCCTTATTTTGGATATTTCTCCCAATG-3’ | 63.5 | 56.1, 58.8, 60.0 |
| PTEN exon 8 | F  R | 5’-GCAAATGTTTAACATAGGTGACAG-3’  5’-GATAACTCAGATTGCCTTATAATAGTC-3’ | 61 | 52.8, 55.3 |
| PTEN exon 9 | F  R | 5’-GTTTAAGATGAGTCATATTTGTGGGT-3’  5’-CAAGTTTATTTTCATGGTGTTTTATCC-3’ | 61.5 | 54.1, 56.9, 57.8 |
| PTEN Intron 8-9 | F  R | 5’-TGATCTTGACAAAGCAAATAA-3’  5’-ACTGCTACGTAAACACTGCTT-3’ | 64 | 55.5 |
| PTEN Microdeletion | Int1-2F  Int3-4R | 5’-CTGCTCCTCTTTACCTTTCTGTC-3’  5’-GTTTTATGGCAAACTCAACTACAGC-3’ | 63 | - |
| N-RAS exon 2 | F  R | 5’-GCTCGCCAATTAACCCTGATTAC-3’  5’-TGGGTAAAGATGATCCGACAAGTGA-3’ | 60.5 | 60.0 |
| N-RAS exon 3 | F  R | 5’-ACACCCCCAGGATTCTTACAGA-3’  5’-TCTTCCCTAGTGTGGTAACCTC-3’ | 63 | 59.2 |
| K-RAS exon 2 | F  R | 5’-GGTACTGGTGGAGTATTTGATAG-3’  5’-CAAAGAATGGTCCTGCACCAGT-3’ | 62 | 58.0 |
| K-RAS exon 3 | F  R | 5’-AGACTGTGTTTCTCCCTTCTCAG-3’  5’-CCCACCTATAATGGTGAATATCT-3’ | 61 | 58.3 |
